# A Multitask Cascading CNN with MultiScale Infrared Optical Flow Feature Fusion-Based Abnormal Crowd Behavior Monitoring UAV †

**DOI:** 10.3390/s20195550

**Published:** 2020-09-28

**Authors:** Yanhua Shao, Wenfeng Li, Hongyu Chu, Zhiyuan Chang, Xiaoqiang Zhang, Huayi Zhan

**Affiliations:** 1 School of Information Engineering, Southwest University of Science and Technology, Mianyang 621010, China; LWF0102@126.com (W.L.); chuhongyu@swust.edu.cn (H.C.); changzy89@gmail.com (Z.C.); xqzhang@swust.edu.cn (X.Z.); 2 Electrical Engineering and Computer Science, Northwestern University, Evanston, IL 60208, USA; huayi.zhan@u.northwestern.edu

**Keywords:** unmanned aerial vehicle (UAV), monitoring, abnormal crowd behavior, multitask cascaded CNN, pyramid L–K optical flow, infrared

## Abstract

Visual-based object detection and understanding is an important problem in computer vision and signal processing. Due to their advantages of high mobility and easy deployment, unmanned aerial vehicles (UAV) have become a flexible monitoring platform in recent years. However, visible-light-based methods are often greatly influenced by the environment. As a result, a single type of feature derived from aerial monitoring videos is often insufficient to characterize variations among different abnormal crowd behaviors. To address this, we propose combining two types of features to better represent behavior, namely, multitask cascading CNN (MC-CNN) and multiscale infrared optical flow (MIR-OF), capturing both crowd density and average speed and the appearances of the crowd behaviors, respectively. First, an infrared (IR) camera and Nvidia Jetson TX1 were chosen as an infrared vision system. Since there are no published infrared-based aerial abnormal-behavior datasets, we provide a new infrared aerial dataset named the IR-flying dataset, which includes sample pictures and videos in different scenes of public areas. Second, MC-CNN was used to estimate the crowd density. Third, MIR-OF was designed to characterize the average speed of crowd. Finally, considering two typical abnormal crowd behaviors of crowd aggregating and crowd escaping, the experimental results show that the monitoring UAV system can detect abnormal crowd behaviors in public areas effectively.

## 1. Introduction

### 1.1. Motivation

With the increase of population and diversity of human activities in recent years, crowd analyses and estimates from videos [1,2]—which have been more frequent in the real world than ever before—have recently attracted increasing interest from the computer vision research community and have become an active research topic, with many applications in maintaining safety and social stability in public places [3,4], intelligent video surveillance [5,6], etc. In the real world, temporary large-scale venues present larger challenges to traditional fixed-video monitoring systems. Due to their high maneuverability and flexible deployment [7], unmanned aerial vehicles (UAVs) could be a promising technology for overcoming the above shortcomings as well as a variety of applications, such as wildlife monitoring and conservation [8], transportation engineering [9], moving target detection [10,11] and monitoring of invasive grasses [12], by combining artificial intelligence and computer vision.

Abnormal event detection involves sensing abnormal activity from surveillance video and then issuing an alarm. In the monitoring of public areas, due to the unpredictability of dangerous types and the complexity of crowd movement, various abnormal crowd events may occur. For different scenarios, abnormal behavior has different manifestations and lack a strict definition [2,5]. Abnormal crowd events can be divided into (1) abnormal individual events and (2) abnormal group events. For individuals, ordinary walking can be understood as normal behavior, while falling can be understood as an abnormal behavior. For crowd, for example, escape caused by fire alarms and aggregate caused by fights are abnormal behaviors.

The understanding of abnormal crowd behavior is the focus of this paper. There are some cases of crowd disasters at mass gathering events: Hillsborough disaster, PhilSports Stadium disaster and the Love Parade disaster [2].

In China, violent incidents of varying types have occurred frequently in recent years, such as the violent terrorist case in Urumqi, Xinjiang in 2009, the terrorist attack on Jinshui Bridge in front of Tiananmen Square in Beijing in 2013, the stampede on the Bund in Shanghai in 2014 and the hacking incident at Kunming Railway Station in Yunnan in 2014, etc. These violent incidents have caused heavy losses to public property and lives. A primary disaster is one aspect; more serious is the resulting stampede, panic, and other secondary disasters. For example, when a crowd stampedes, and many may be crushed or trampled underfoot. These secondary disasters are mainly manifested as aggregating and escaping. As a result, aggregating and escaping are two typical representatives of abnormal crowd behavior in the field of public security.

In this paper, the factors of average speed and density are used to judge abnormal crowd behavior in the field of public security. In general, when an abnormal crowd behavior occurs in a public area, it is often accompanied by an increase or decrease in crowd density, and the speed of crowd movement suddenly increases or decreases. For example, when a terrorist attack or a fire alarm occurs, the crowd will appear to run around, and the number of people in the video surveillance will drop. When congestion or trampling is imminent, it is often accompanied by the phenomenon of increasing crowd density and decreasing crowd speed. Therefore, in the field of public security, it is very important to carry out dynamic density-change detection and speed-change detection in public areas to judge the abnormal behavior of crowds.

However, abnormal-crowd-behavior monitoring from infrared images obtained from UAV poses many challenges such as: (1) effective mechanism design and monitoring strategy of UAV meeting detection and recognition requirements, (2) effect of natural background and noise in infrared images, fuzzy edges of infrared aerial objects, making it difficult to segment and label person objects in natural backgrounds, (3) large variations in the scale and appearance of aerial person objects from severe perspective distortion of the scene and the relative movement between human objects and the onboard camera and (4) finding a reasonable crowd motion criterion for abnormal behavior monitoring.

### 1.2. Literature Review

Many researchers have focused on single-pedestrian detection [13,14,15], crowd counting and analysis [3,5,6,16,17,18] and UAV-based computer-vision applications.

Some examples of this include monitoring wildlife [8], invasive grasses and vegetation [12], close-range interaction [19], detecting roads [20], vehicles and pedestrians [21], etc.

Features act as a key factor in the challenge of pedestrian detection. According to the characteristics of the feature extraction for pedestrian detection, there are two methods: sliding window approaches (also denoted as traditional approaches) and deep learning-based methods. The former, which is typically represented by histogram of oriented gradient (HOG) and discriminative part-based model (DPM) [14], appears promising for low to medium-resolution settings, under which segmentation or key-point-based methods often fail [13]. In the last few years, deep-learning and in particular, convolutional neural networks (CNN) have emerged as the state of the art in terms of accuracy for pedestrian detection—often outperforming the previous gold standards by a large margin [15,22]. To exploit more contextual information, a multitask cascade CNN (MC-CNN) framework was proposed for thyroid nodule recognition in [23].

Crowd analysis is a subdomain of human-activity recognition. Based on the reviews and analysis in [3,5,18,24], existing methods for crowd counting and estimate are categorized into the following three categories: (1) detection-based methods, (2) features-regression-based methods and (3) density-estimation-based methods. For example, the change of energy-level distribution [1] and Bayesian risk kernel density [25] have been proposed. The earlier detection-based methods, which are vulnerable to threats of occasion, illumination intensity, fluctuating of background and noise, are often based on sliding-window approaches. The features-regression-based methods are used extensively recently [3].

Several good results in computer vision and other fields have been obtained by using deep-learning-based means in recent years. Crowd counting and estimation is no exception [3,5,6,26]. Su et al. present a coherent long short-term memory (cLSTM) network to capture nonlinear crowd dynamics by learning from an informative representation of crowd motions [26]. Sindagi et al. proposed a novel system of end-to-end cascaded CNNs to jointly learn crowd-count classification and density-map estimation; joint training is performed in an end-to-end way [6]. The multicolumn CNN model, which allows the input image to be any arbitrary size or resolution, is presented in [3]; a true-density map is accurately computed based on geometry-adaptive kernels that also do not need to know the perspective map of the input image. In [27], an attention-injective deformable CNN for crowd understanding was proposed to address the accuracy degradation problem of highly congested noisy scenes.

More comprehensive analyses and survey of different crowd counting and estimate approaches can be found in [5,18,28].

Commercial delivery by UAVs is expected to become a widespread service in the near future. The actual operation scenarios of UAV are often complex; any safety problem, e.g., possibility of collision between UAVs, drone loss of control, etc.., must be avoided in actual deployment [29]. Therefore, an unmanned aircraft system must incorporate conflict detection and resolution (CDR) methods [30,31].

Abnormal crowd-behavior monitoring focuses on identifying abnormal activity or emergency situations in crowd scenes. Because of severe occlusions, extreme clutter, large variations in scale and appearance of the objects in crowded scenes, conventional methods without special considerations are not appropriate. In addition, visible-light-based methods are often greatly influenced by environment. At the same time, temporary large-scale venues present higher challenges to the common fixed-video monitoring systems. This research aims to address the above challenges by proposing an abnormal-crowd-behavior monitoring system, which focuses on the two typical abnormal crowd behaviors of aggregating and escaping by using low-resolution thermal images recorded by the onboard thermal infrared cameras in a UAV system. A fusion-based approach, i.e., multitask cascading CNN (MC-CNN) and multiscale infrared optical flow [32] (MIR-OF), is employed to detect abnormal behavior in crowd scenes.

### 1.3. Contributions

Contributions and innovations of this paper are summarized as follows:

(1) Since there are few published infrared-based aerial abnormal-behavior datasets obtained from UAV, we assembled a new infrared aerial dataset named the IR-flying dataset that includes sample pictures and videos in different scenes of public areas.

(2) A fusion algorithm is proposed. Accurate crowd density is obtained from a MC-CNN. MIR-OF is applied to track the motion corners [32]; the motion vectors of the motion corner points in two consecutive frames is obtained for the average velocity.

(3) A UAV system was designed and built, and all the algorithms were transplanted into the onboard Jetson TX1. The experimental results show that the monitoring UAV system can detect abnormal crowd behavior in public areas effectively.

### 1.4. Organization

The rest of the paper is organized as follows: Section 2 describes the algorithm research and the system design, involving four parts: the hardware system design and realization of the abnormal-crowd-behavior-monitoring UAV, using MC-CNN for crowd-density estimation, MIR-OF-based crowd-motion estimation and fusion-based abnormal crowd behavior recognition. Experimental results are analyzed in Section 3. Finally, conclusion remarks are given in Section 4.

## 2. Algorithm Research and System Design

In this section, the details of the proposed system are described. The main content is as follows: Section 2.1 describes the hardware system design and algorithm realization of abnormal-crowd-behavior monitoring UAV. MC-CNN-based crowd-density estimation is proposed in Section 2.2. In Section 2.3, the MIR-OF is proposed for the average velocity. Section 2.4 presents the detailed decision flow for abnormal crowd behavior recognition.

### 2.1. System Architecture

**Figure 1:**
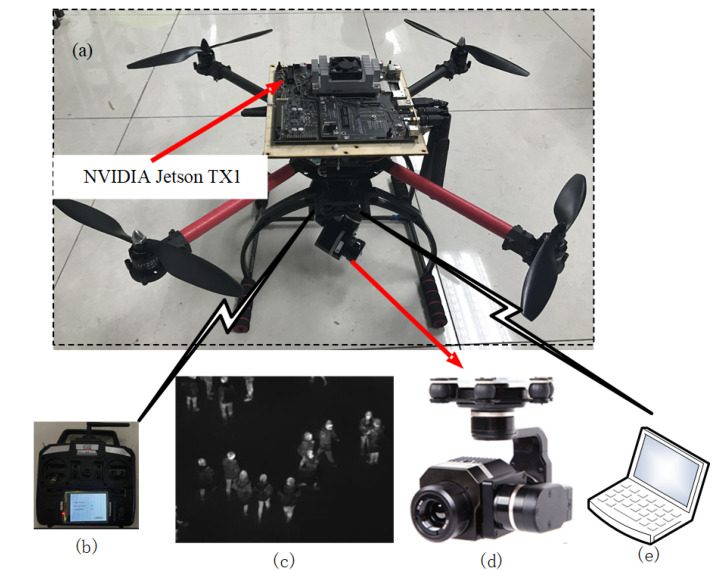
Schematic diagram of monitoring unmanned aerial vehicles (UAV). (**a**) UAV with an infrared camera; (**b**) remote control; (**c**) representative image in our new infrared-based abnormal crowd behavior dataset; (**d**) FLIR TAU2-336 infrared thermal imager; (**e**) ground control station.

The entire UAV system can be divided into an infrared camera, remote control, and ground control station (Figure 1). In this section, we briefly describe the system architecture of the monitoring UAV owing to space reasons. Overall, picture of monitoring UAV is shown in Figure 1a. STM32F427VIT6 was adopted as the core of flight control system. The infrared vision system consists of an infrared camera FLIR TAU2-336 and a Jetson TX1 for image processor, which is shown in Figure 1a,d. The ground control station, which is based on a well-known open source software QGroundControl, communicates with the UAV via MAVLink.

**Figure 2:**
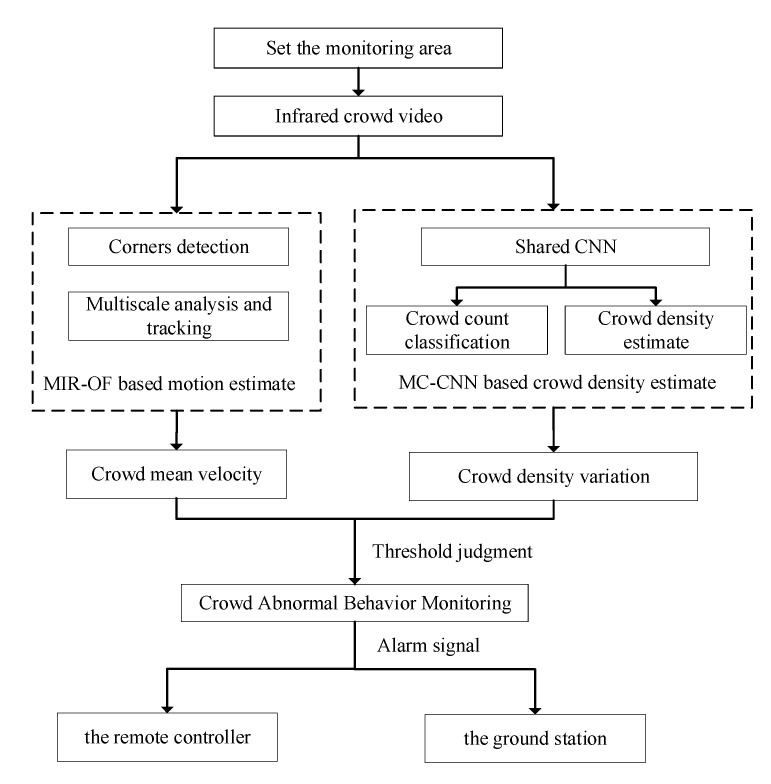
Framework of the proposed UAV system.

Figure 2 presents a brief overall flowchart of abnormal-crowd-behavior monitoring system. The basic flow of the application operation is as follows: (1) The monitoring area was designated by remote control; (2) a thermal infrared imager (FLIR TAU2-336), which was installed on our UAV, was used for taking pictures of the designated outdoor area; (3) the MC-CNN-based crowd-density estimation and crowd-motion information, which was settled through MIR-OF average velocity method-based was obtained by using high-performance embedded system with NVDIA Jetson TX1. Finally, a fusion-based approach, i.e., crowd density and crowd mean velocity, was employed to detect abnormal behavior in crowd scenes. Whether the abnormal crowd behavior occurs was determined by comparing the value of the descriptors with their corresponding threshold. When the fusion descriptors were determined to be abnormal, an alarm prompt was raised. Several actual experimental results showed that the monitoring UAV system could effectively detect abnormal crowd behavior in public areas.

### 2.2. MC-CNN-Based Crowd-Density Estimation

CNN has been actively researched over the past several years. Inspired by the success of the related multitasking cascade CNN [6,33,34], and taking into account the computing power, storage space and power consumption of the embedded platform Jetson TX1, a two-stage crowd-density estimate method was adopted in this research to count people accurately.

**Figure 3:**
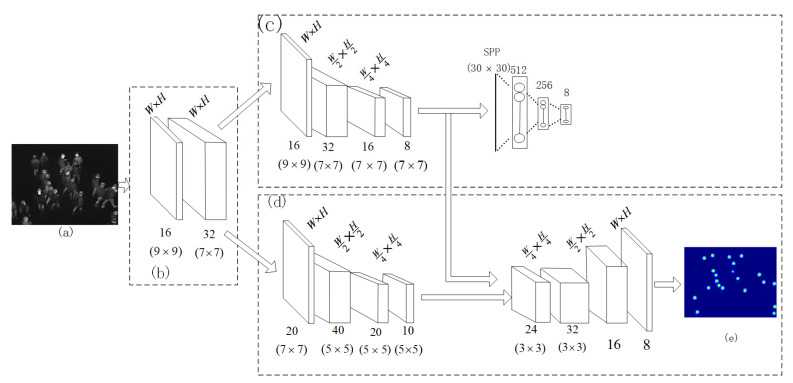
Schematic diagram of the MC-CNN. (**a**) Input infrared image; (**b**) shared CNN; (**c**) first stage; crowd-count classification; (**d**) second stage; crowd-density estimate; (**e**) crowd-density estimate map corresponds to the input infrared image.

A schematic diagram of the MC-CNN is presented in Figure 3. The brief workflow was as follows: With an aerial infrared image as shown as Figure 3a as the input, the feature maps were obtained by using the shared CNN as presented in Figure 3b. Then, the shared feature maps were used by crowd-count classification and density-estimate stages, which are shown in Figure 3c,d, respectively.

**Figure 4:**
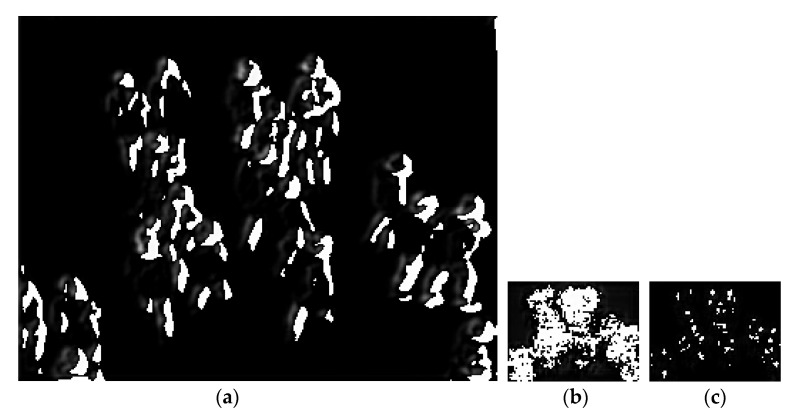
Representative feature maps in the MC-CNN. (**a**) Shared CNN; (**b**) crowd-count classification stage; (**c**) first stage of crowd-density estimate.

Visualizing features to gain intuition about the CNN is common practice [35]; representative feature maps in the MC-CNN are presented in Figure 4. The resolution of Figure 4a (one of 32 instances), Figure 4b (one of eight instances) and Figure 4c (one of 10 instances) is 336 × 256, 84 × 64 and 84 × 64, respectively. As seen in Figure 4, the projections from each layer show the hierarchical nature of the features in the network and show its invariance to input image as shown in Figure 3a. Note that Figure 4b provides more global information than will affect crowd-count classification. Correspondingly, the individual information that is more useful for counting is shown in Figure 4c. These indicate that network training is effective and consistent with what we expect from our projections.

The specific size of the feature map in Figure 3 is marked in detail. The following mainly discusses the related processing flow and some specific details for the training.

#### 2.2.1. Crowd-Count Classification

In the field of machine-learning, the directionality of classification problems can be improved by using more distinct and meaningful classification labels. Therefore, it is easier to divide the crowd into some special rough groups than to directly classify or regress the entire population count range.

According to the characteristics of the scene in a university, in this paper, a classifier is built in the crowd-count classification stage and performed the task of dividing the crowd into eight groups. As shown in Figure 3c, the final layer in the first stage contains eight neurons.

In this stage, cross-entropy error is used and defined as follows:

(1) $$L_{c}=-\frac{1}{N}\overset{N}{\underset{i=1}{\sum }}\overset{M}{\underset{j=1}{\sum }}\left[\left(y^{i}=j\right)F_{c}\left(X_{i},\Theta_{c}\right)\right]$$

where, *N* is the number of training samples, *M* is the total number of classes (*M* = 8) as shown in Figure 3c. *y^i^* is the ground truth class, $F_{c}\left(X_{i},\Theta_{c}\right)$ is the classification output, *X_i_* is the *i-th* training sample and $\Theta_{c}$ represents the network parameter in this stage.

#### 2.2.2. Density-Map Estimation

The feature maps obtained from the shared layers, which are shown in Figure 3b, are processed by the density-map estimation stage that consists of 4 convolutional layers with a parametric rectified linear unit (PReLU) activation function after every layer as shown in Figure 3d.

The loss function for this stage is defined as follows:

(2) $$L_{d}=\frac{1}{N}\overset{N}{\underset{i=1}{\sum }}∥F_{d}\left(X_{i},C_{i},\Theta_{d}\right)-D_{i}{∥}_{2}$$

where, $F_{d}\left(X_{i},C_{i},\Theta_{d}\right)$ is the estimated density map, *X_i_* is the *i-th* training sample, *C_i_* are the feature maps obtained from the last convolutional layer of the crowd-count classification stage, *D_i_* is the ground-truth density map, and $\Theta_{d}$ represents the network parameters of this state. The entire cascaded network is trained using the following overall loss function:

(3) $$L=\lambda L_{c}+L_{d}$$

where, *λ* is the weighting factor. Experiments show that the *L_c_* has virtually less performance impact on the overall loss function by itself, therefore in this paper, we choose *λ* = 0.00001 after multiple validation.

#### 2.2.3. The Training of MC-CNN

In this paper, the CNN training platform is the HP OMN notebook, which has 2.5 GHz CPU and 16 GB memory, and the graphics card is NVIDIA GeForce GTX 1050Ti powered by CUDA 10.1 using PyTorch 1.4.

**Figure 5:**
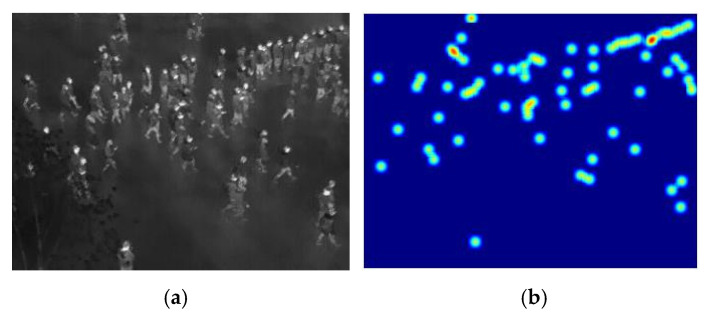
Density map obtained by geometric adaptation of Gaussian kernel. (**a**) Input image; (**b**) corresponding density map.

The performance of the CNN model is determined by the calibration quality of the target crowd in the training data. This section describes how to convert the labeled human head into a density map. In order to adapt the crowd-density map to different perspectives or different head size in crowded images, the geometry-adaptive Gaussian kernel density mapping method [3] is adopted in this paper can be expressed as:

(4) $$D\left(x\right)=\overset{N}{\underset{i=1}{\sum }}\delta \left(x-x_{i}\right)*G_{\sigma_{i}}\left(x\right)$$

where, *x_i_* is the location of the head in the image. $\delta \left(x-x_{i}\right)$ is the impulse function for the position of the human head in the image, *N* is the total number of the head. Figure 5 illustrates the density map results obtained using the proposed method.

The detailed training process was as follows:

(1) Preparation of the training set. In this paper, the number of training samples was 607 and the test samples were 260. The details of the train dataset are described in Section 3.1;

(2) Data augmentation. Data augmentation helps prevent the network from overfitting and memorizing the exact details of the training images. Specifically, the input picture could be rotated the scope of ±5°;

(3) Parameter initialization. The learning rate was 0.00001 and momentum was 0.9;

(4) Training of the model. We tested the training time in different environments. The training time was 30 h with the GPU. The training time was 168 hours without the GPU. The test accuracy of the two models was similar.

### 2.3. MIR-OF-Based Crowd-Motion Estimate

**Figure 6:**
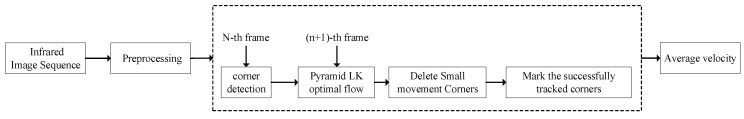
Crowd-motion estimate.

Crowd-motion information is very important for abnormal crowd behavior analysis. The details for our corner detection and tracking process is shown in Figure 6.

In this section, we combine Shi–Tomasi corner detection and pyramid LK optical flow method to estimate the crowd-motion information. Based on this, the average moving speed of all corner points is calculated to estimate the average of the crowd.

#### 2.3.1. Corners Detection and Multiscale Analysis and Tracking

**Figure 7:**
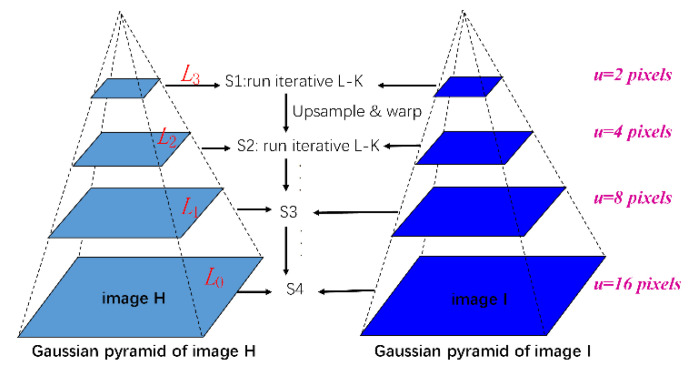
Three-level pyramids of two continuous frames.

In order to avoid the effect of corner point shift caused by small motion or environmental interference in the background, the interference corner points in the background needed to be removed from the detected set of initial corners. After this, more effective crowd-motion information can be extracted. In this paper, the multiple scale method, which was proposed by Shi and Tomasi, was adopted for the movement information of the crowd in the monitoring scene [36,37,38]. Figure 7 shows three-level pyramids of two frames *H* and *I*. These four steps represent S1, S2, S3 and S4, respectively. Steps 3 and 4 were similar to Step 2.

The main steps, each of which is explained in detail, were as follows:

(1) Two consecutive frames of images *H* and *I*, were obtained at the same time, using corner detection on frame *H* and the successfully detected corners *C1* from frame *H* were regarded as the initial point of the pyramid LK Optical flow for tracking;
(2) The successfully detected and tracked corners from frame *I* were recorded as *C2*;
(3) The amplitude of velocity were calculated, written *mag*, of the corresponding corner between *C1* and *C2*;
(4) We determined if the velocity amplitude of each corner in mag was greater than the small motion threshold. If it was greater than the small motion threshold, the speed information of the corner was preserved or vice versa.

**Figure 8:**
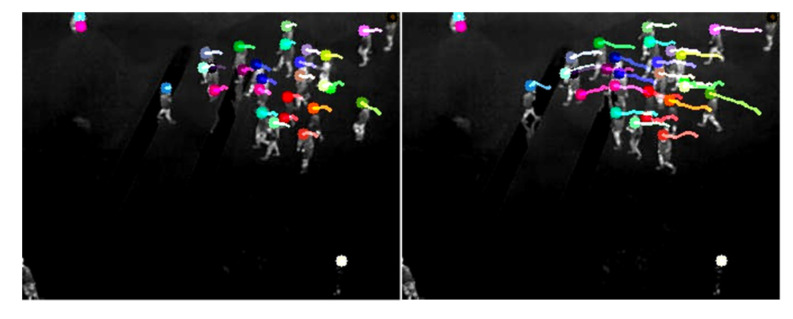
Result of motion corners detection and tracking.

Further details for the pyramid method can be found in [19]. The experiment results of motion corners detection and tracking are shown in Figure 8. The experimental results show that the multiple scale Shi–Tomasi corner tracking was more stable.

#### 2.3.2. Average Velocity

Under normal circumstances, this fluctuation range of crowd velocity is small, when the abnormal crowd behavior happens, such as the crowd aggregating and crowd escaping and other abnormal behavior, the average speed of the crowd suddenly become large or suddenly small, therefore, the average velocity of the crowd can be as select a reasonable descriptive operator for abnormal behavior.

For two continuous frame image, the optical flow information, written as ${\left(v_{x},v_{y}\right)}^{T}$, of the moving corner is obtained by the pyramid-based L–K optical flow [32]. Thereby, the speed of corner can be calculated as:

(5) $$v=\sqrt{v_{x}^{2}+v_{y}^{2}}\times fps$$

where, *v_x_* and *v_y_* are the partial velocity of the optical flow with respect to the x-axis and the y-axis, respectively. The fps represents the frames per second. Moreover, *fps* = 7, which is the frame rate of the onboard infrared camera.

The average velocity of frame x, written *v*(*x*), can be defined as:

(6) $$v\left(x\right)=\frac{1}{n}\times \overset{n}{\underset{i=1}{\sum }}v_{i}$$

where *n* indicates the number of moving corners detected and the *v_i_* represents the motion velocity of the *i*-th corner point.

### 2.4. Decision Flow for Crowd Abnormal Behavior

In order to distinguish the normal behavior and abnormal behavior of the crowd accurately and effectively, it is necessary to find the descriptive feature with significant changes in the two cases of normal and abnormal behavior. In this study, a abnormal crowd behavior detection method combining CNN-based crowd-density characteristics and crowd speed characteristics is used.

Consider velocity factor and density factor together, according to the guidance of domain experts, the criteria for determining abnormal behavior can be summarized as shown in Table 1.

Through the above statistical analysis, according to Table 1, the normal behavior of the crowd and the abnormal behavior of the crowd can be classified based on some detailed criteria.

## 3. Experimental Results and Validation

In this section, we demonstrate the experiment results of our outdoor autonomous monitoring UAV based on crowd-density characteristics, corners detection and multiple scale pyramid optical flow. Our evaluation UAV system consists of an embedded NVDIA Jetson TX1 with 256 NVIDIA CUDA® cores and Samsung 4 GB 64-bit LPDDR4 Memory, running Ubuntu16.04 and an implementation of fusion feature-based crowd-motion estimation by using Python 3.6 and OpenCV 2.4.13.

### 3.1. Our Self-Built Data Set: IR-Flying Dataset

**Figure 9:**
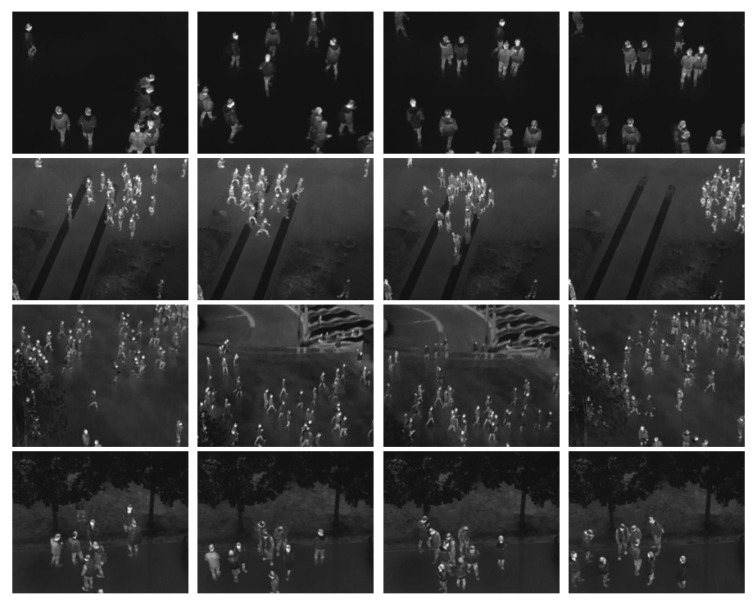
Representative images of our new crowd dataset. The samples in rows 1 to 4 indicate near the building, intersections, squares, and roads, respectively.

Most currently available crowd datasets are based on visible light. Only the OTCBVS dataset includes some infrared images. However, the dataset does not include abnormal crowd behavior. This paper creates a crowd-behavior dataset based on aerial infrared images in different scenarios. This is named the IR-flying dataset. In addition, the abnormal behaviors of both aggregating and escaping in different typical scenarios are simulated. The detailed information of the dataset is shown in Table 2. Figure 9 shows some representative samples of this dataset.

Abnormal behavior is closely related to specific scenes. Combined with specific event instances and scenes, it is easier to understand abnormal than normal crowd behavior. Detailed information concerning abnormal behavior and the typical scenarios in which they occur is shown in Table 3.

In this section, the experimental results of crowd-density estimation and the results of crowd-motion estimation are analyzed, respectively. Then, the abnormal behavior of the crowd is detected by combining the crowd-density characteristics and the crowd movement characteristics, and the experimental results are analyzed.

### 3.2. Experiments for Crowd Abnormal Behavior Monitoring

This paper simulates two typical abnormal crowd behaviors of aggregating and escaping in two scenarios. Scene #1 represents a crossroad, while Scene #2 is near a building. Here we simply refer to buildings. The average movement speed of two consecutive frame can be obtained according to the formula (6) with the height of the UAV is 20 m.

When the average movement speed of the crowd becomes larger or smaller, the crowd movement alarm is carried out.

**Figure 10:**
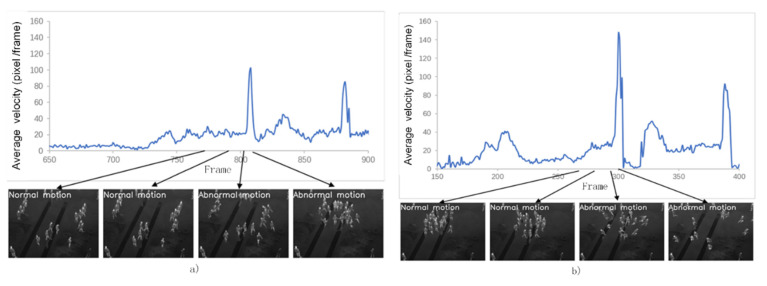
Crowd behavior recognition. (**a**) Crowd-aggregating motion detection, (**b**) crowd-escaping motion detection.

After filtering, when the absolute value of the average velocity difference of two consecutive frames is greater than the threshold *th*, it is considered that the abnormal movement of the crowd is true, and the system alarm is provoked. The threshold is an indirect characteristic, which is calculated from positive and negative samples suggested by field experts in practical experiments. Therefore, according to the opinions of experts and the experimental verification of the average velocity, this paper sets the threshold *th* equal to 10 (height = 20 m). The results of the population aggregation movement are shown in Figure 10.

Using Figure 10a as an example, “normal” indicates that the crowd is behaving normally. Likewise, "abnormal" indicates that the crowd is behaving abnormally.

As the crowd gathers, the speed of the crowd suddenly becomes larger. The system starts to alarm at the 806*th* frame. Then the crowd walks around at random. The average movement speed of the crowd tends to be stable, so the system does not alarm. As the crowd gathers again, the system starts to alarm at the 880*th* frame, then the crowd walks around, the crowd moves normally, and the system does not alarm. Based on similar criteria, as shown in Table 1, the system starts to alarm at the 300*th* and 387*th* frame.

Overall, our UAV can correctly detect the number of frames with the crowd anomaly and identify the crowd anomaly behavior of the crowd gathering and the crowd scattered.

**Figure 11:**
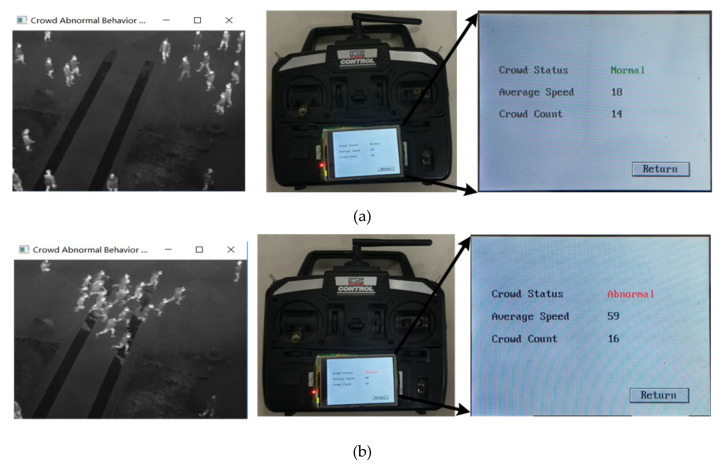
Crowd-status information of crossroad scene. (**a**) Normal; (**b**) abnormal.

**Figure 12:**
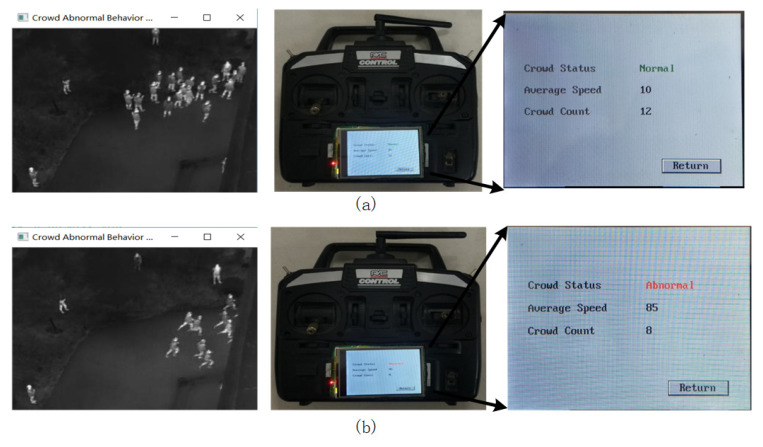
Crowd-status information of the scene close to the building. (**a**) Normal; (**b**) abnormal.

The Jetson TX1 sends the crowd-status information to the flight control system through the serial port. The operator can obtain the crowd-status information from the handheld remote control, and the ground station system can obtain real-time image information through the image-transmission module. The crowd-status information acquired by the handheld remote controller from the crossroad scene and the scene close to the building is shown in Figure 11 and Figure 12, respectively.

It can be seen from Figure 11 and Figure 12 that the ground operator can obtain the state information of the crowd in real time—including the behavior state of the crowd, the number of people and the average speed of the crowd.

In general, the results predicted by the algorithm are divided into the following four cases: true positive (TP), true negative (TN), false positive (FP) and false negative (FN). The meaning is shown in Table 4.

In order to further verify the reliability and correctness of the system, this paper uses the recall rate (recall), precision (precision), accuracy (accuracy) and F1 score (F_1_) in the information retrieval field to statistically analyze the test results and evaluate it as an algorithm.

The specific definition are as follows:

(7) $$Precision=\frac{TP}{TP+FP}$$

(8) $$Recall=\frac{TP}{TP+FN}$$

(9) $$Accuracy=\frac{TP+TN}{TP+TN+FP+FN}$$

(10) $$F_{1}=\frac{2\times TP}{2\times TP+FP+FN}$$

Precision reflects the model’s ability to distinguish negative samples, Recall reflects the model’s ability to recognize positive samples and F1 score is a combination of the two and the model with high F1-score is more robust. The emphasis on precision and recall varies in different scenarios. In the field of public safety, it is more desirable to miss as little as possible the real abnormal behavior of the crowd. Test and analyze the two actual scenarios. The experimental results are shown in Table 5. In addition, the average single anomaly detection time was counted using the method of this study, as shown in Table 6.

In this paper, the average speed and density factor were used to judge the abnormal behavior of the crowd. Crowd-density estimation is an important part of detection. In the research process, our MC-CNN-based method was superior to the typical multicolumn convolutional neural network (MCNN). It can be seen from Table 3 that the surrounding environment changes had little effect on the abnormal behavior detection of the crowd of Scene #1 and Scene #2. The density of the population in Scene #1 and Scene #2 was different. Specifically, the precision rate and recall rate were quite different in Scene #1 and Scene #2. However, the F1 scores of different scenes were very close, indicating that our model had certain robustness for different scenes. Moreover, the correct rate of the abnormal crowd behavior detection system designed in this paper could reach more than 90%, which satisfies the detection requirements of abnormal crowd behavior of the actual scene.

It can be seen from Table 6 that the average time of crowd abnormality detection on Jetson TX1 is about 0.22 s, which satisfies the real-time monitoring needs of the actual scene.

## 4. Conclusions

This paper proposes an approach to detect abnormal crowd behaviors in low-resolution aerial thermal infrared images. The proposed infrared abnormal-crowd-behavior monitoring method consists of two parts: (1) the MC-CNN is designed to estimate the crowd density; (2) the MIR-OF is designed to characterize the average speed of crowd. Utilizing the flexibility of the UAV and the characteristics of infrared imaging, our system can monitor both bright and dark crowd objects in either daylight or at night. Furthermore, since there are no published infrared-based aerial abnormal crowd behavior datasets obtained from UAV, we self-built a new infrared aerial dataset named the IR-flying dataset, which includes sample pictures and videos in different scenes of public areas. Finally, aiming at two typical abnormal crowd behaviors of crowd aggregating and crowd escaping, the experimental results show that the monitoring UAV system, which is equipped with infrared (IR) camera and Nvidia Jetson TX1, can achieve the detection of abnormal crowd behavior in public areas effectively.

The method in this paper is aimed at crowd behavior and cannot effectively detect a single person’s fast moving or abnormal behavior. However, as individual abnormal behavior such as violent attacks can lead abnormal crowd behavior, such as escaping, our system can effectively detect abnormal crowd behavior in the field of public security.

Due to the low contrast of the infrared image, the drone moves with the crowd target, combining visible light images. Developing better algorithms for individual and crowd behavior analysis or cooperative monitoring with multiple UAVs [39] is one of the main working directions in the future.

## Figures and Tables

**Table 1:** Event classification.

Velocity Factor	Density Factor	Normal/Abnormal
Becomes larger	Becomes smaller	Abnormal
Becomes smaller	Becomes larger	
Becomes larger	Becomes larger	
Becomes larger	Constant	
Constant	Becomes larger	
Becomes smaller	Becomes smaller	Normal
Becomes smaller	Constant	
Constant	Becomes smaller	
Constant	Constant	

**Table 2:** Details of the self-build datasets.

Attribute	Attribute Values
Resolution	336*256
Scene	4
Image num	970
Person num	16,000
Frame rate	7

**Table 3:** Scenarios applied for each type of crowd behavior.

Type of Behavior	Scenarios
#1: Aggregating	Traffic congestion Demonstration Trampled underfoot Fight
#2: Escaping	Terrorist attack Fire alarm Earthquake

**Table 4:** Algorithm prediction result and its meaning.

Prediction Result	Meaning
TP	Prediction is abnormal, the actual is abnormal.
TN	Prediction is normal, the actual is normal.
FP	Prediction is abnormal, the actual is normal.
FN	Prediction is normal, the actual is abnormal.

**Table 5:** Actual scene experiment results.

Scene	TP	TN	FN	FP	Accuracy	Precision	Recall	F1-Score
#1: Intersections	27	1103	6	7	98.86%	79.41%	81.81%	80.59%
#2: Buildings	40	1443	5	15	98.66%	72.72%	88.88%	79.99%

**Table 6:** Average single anomaly detection time.

Scene	Detection Time/s
#1: Intersections	0.224
#2: Buildings	0.219

## Abbreviations

The following abbreviations are used in this manuscript:

CNN	Convolutional neural network
L–K	Lucas–Kanade
MC-CNN	Multitask cascading CNN
MIR-OF	Multiscale infrared optical flow
UAV	Unmanned aerial vehicles
TP	True positive
TN	True negative
FP	False positive
FN	False negative
